# Prevalence of anemia and its associated factors among pregnant women attending antenatal care follow up at Wollega University referral hospital, Western Ethiopia

**DOI:** 10.1186/s40834-020-00130-9

**Published:** 2020-10-09

**Authors:** Gemechu Kejela, Aga Wakgari, Tariku Tesfaye, Ebisa Turi, Moa Adugna, Netsanet Alemu, Latera Jebessa

**Affiliations:** 1 Department of Public Health, Institute of Health Sciences, Wollega University, Nekemte, Ethiopia; 2 School of Medicine, Institute of Health Sciences, Wollega University, Nekemte, Ethiopia

**Keywords:** Anemia, Pregnant women, Hemoglobin

## Abstract

**Background:**

Even if anemia is a worldwide public health problem affecting numerous people in all age groups, particularly the burden of the problem is higher among pregnant women. Anemia is estimated to contribute to more than 115,000 maternal deaths and 591,000 prenatal deaths globally per year. Maternal mortality is the prime health indicator in any society. Therefore, determining the prevalence of anemia and assessing its associated factors among pregnant women might help for the intervention of the problem.

**Objective:**

The objective of this study was to determine the prevalence of anemia and its associated factors among pregnant women attending Antenatal Care (ANC) at Wollega University Referral Hospital, Western Ethiopia.

**Methods and materials:**

Institution based cross-sectional study was conducted at antenatal care (ANC) departments of Gynecology and Obstetrics, and MCH at Wollega University Referral Hospital from July 15–22, 2019. A systematic random sampling technique was used to select the study unit. The data were collected using questionnaires, physical examination, and laboratory investigation. After collection, the data were entered using Epi Data version 3.1 and analyzed using SPSS version 20 statistical software. Bivariate and multivariate logistic regression analysis was performed to identify predictors of anemia. Finally, the result was presented using text, tables, and charts.

**Results:**

The overall prevalence of anemia using a cut off level of hemoglobin < 11 g/dl (< 33% Haematocrit) was 51 (17.8%). Out of all anemic pregnant women, 19 (37.25%) were mildly anemic, 24 (47%) were moderately anemic and 8 (15.68%) were severely anemic. Multivariable logistic regression analysis revealed that Birth interval of less than 2 years (AOR = 2.56 CI [2.84–4.52]), history of malarial attack in the past 12 months (AOR = 2.585 CI [1.181–5.656]) and engaging into daily laborer occupation (AOR = 8.33 CI [2.724–25.497]) showed significant association with maternal anemia.

**Conclusions:**

The prevalence of anemia among pregnant women in this study is high. Having a birth interval of < 2 years, having a history of malarial attack in the past 12 months, and being engaged in daily laborer occupation were factors associated with anemia among pregnant women. Thus, contraceptive methods and information to space children, information, and services to prevent malaria and economically empowering women is needed to prevent anemia among pregnant women in the study area.

## Background

Anemia is defined as a condition in which there is less than the normal hemoglobin level in the body, which decreases the oxygen-carrying capacity of red blood cells to tissues. World Health Organization (WHO) definitions for anemia differ by age, sex, and pregnancy status as follows: in children 6 months to 5 years, anemia is defined as a Hemoglobin level < 11 g/dl; in children 5–11 years, Hemoglobin < 11.5 g/dl; in adult males, Hemoglobin < 13 g/dl; in non-pregnant women, Hemoglobin < 12 g/dl and in pregnant women Hemoglobin < 11 g/dl [1].

Anemia could be classified as mild, moderate, and severe. The Hemoglobin 10.0 g/dl for each class of anemia in pregnancy is considered mild. The hemoglobin level from 7 to 9.9 g/dl is considered as moderate and if < 7 g/ dl, it is considered as severe. Anemia is considered as an indicator of both poor nutrition and health status [2, 3].

Anemia is one of the most widespread public health problems, especially in developing countries. It impairs cognitive development, reduced physical work capacity, and in severe cases increased the risk of mortality particularly during the prenatal period. During pregnancy, approximately 75% of all anemia diagnosed are due to iron deficiency. Furthermore, WHO considers that women in developing countries may be pregnant for as much as one-half of their reproductive lives and therefore are at increased risk of anemia during this time [4]. About 20% of pregnant women suffer from anemia, and most of the cases are iron deficiency, folic acid deficiency, or both. The administration of iron and folic acid to pregnant women is a divisive issue, and the guiding principle regarding this remedy varies among countries [3].

Globally, anemia is the most common complication in pregnancy. The World Health Organization estimates that more than 40% of non-pregnant and over 50% of pregnant women in developing countries are affected. The majority of the cases occur in sub-Saharan Africa and Southeast Asia [5].

Anemia in pregnancy leads to premature births, low birth weight, fetal impairment, and infant deaths. It also reduces the productivity of women. The reduction in women’s productivity places an economic burden on families, communities, and societies. Recently, the mental impairment of children who were anemic at the very beginning of their life has been reported. All of those showed the necessity of a special control program for anemia in vulnerable populations [6].

Food-based approaches to increase iron intake through food fortification and dietary diversification are important, sustainable strategies for preventing iron deficiency anemia in the general population. In settings where iron deficiency is not the only cause of anemia, approaches that combine iron interventions with other measures are needed. Strategies should include addressing other causes of anemia and should be built into the primary health care system and existing programs [7, 8].

In Ethiopia, the Federal Ministry of Health has laid out a policy on iron supplementation to all women attending antenatal clinics [9]. As a result, pregnant women attending antenatal clinics are routinely put on iron supplementation in their second through to the third trimester of pregnancy.

Despite all the above efforts, the burden of disease remains high as determined by anemia related to fetal and maternal mortality and morbidity. As a result, knowing the prevalence of anemia and its associated factors in pregnant women is fundamental for the planning and execution of effective interventions by health authorities. So, the main aim of this study was to assess the prevalence of anemia and its associated factors among pregnant women attending the ANC clinic of Wollega University referral hospital and the results of this study will help to put in place policies to effectively investigate and manage anemia in pregnancy and thereafter to reduce the burden of disease.

## Methods and materials

An institution-based quantitative cross-sectional study was carried out from July 15–22/2019 in Nekemte administrative city, at Wollega University Referral Hospital among 286 respondents. Nekemte town is the capital city of the East Wollega zone and it is located at 331 Km to the west of Addis Ababa. Its altitude ranges from 1960 to 2170 above sea level and the total surface area of the town is 5480 Km^2^. The climatic condition of the town is Woina dega with an annual environmental temperature range of 14–26 degrees Celsius. The total population of the town as 2018 data is 127,380 from which 65,002(51.03%) are males and 62,378 (48.97%) are females. Pregnant women accounts for 4420(3.47%) and non-pregnant women accounts for 23,731 (18.6%). Regarding the health facilities of the town, there are two governmental Hospitals, two governmental Health centers, 21 medium clinics, 26 lower clinics, 12 pharmacies, and 26 drug stores [10].

Wollega University Referral Hospital is a teaching hospital in western Ethiopia serving millions of people. The hospital has different departments, of which the obstetrics and gynecology department is one of the main departments. This department gives ANC, pregnancy-related problem diagnosis and management, delivery, and post-delivery service.

All pregnant women attending ANC service at Wollega University Referral Hospital were the source population and all pregnant women attending ANC Service at Wollega University Referral Hospital who fulfilled the inclusion criteria and available during the data collection period were the study population for this study. All pregnant women who attended antenatal clinics at Wollega University Referral Hospital during the study period were included in the study and seriously ill patients due to other medical conditions, unable to respond, and mentally ill pregnant women were excluded from the study.

### Sample size determination and sampling procedures

The sample size was calculated by using a single population proportion formula (n = (Z ⍺ /2)^2^ p (1-p)/d^2^), considering the following assumptions;

Z⍺/2 (significance level) at 0.05 = 1.96, d (margin of error) = 5% and P (proportion of anemia) in reproductive age group women in western Ethiopia =21.6% [11]. After adding a 10% nonresponse rate, the final sample size becomes 286. Finally, the study respondents were selected from pregnant women who visited ANC during the study period by using simple random sampling.

### Measurements

In this study, anemia in pregnancy is defined as a hemoglobin level below 11 g/dl during the first and third trimester and 10.5 g/dl during the second trimester of pregnancy. Mild anemia is defined as a hemoglobin level from 10 to 10.9 g/dl., moderate anemia is defined as a hemoglobin level from 7 to 9.9 g/dl, and severe anemia was defined as a hemoglobin level of < 7 g/dl.

### Data collection instruments, procedure, and quality assurance

Data were collected using an interviewer-administered structured questionnaire, physical examination, and laboratory investigation. The questionnaire was prepared from similar studies conducted before and modified based on the local context. It was prepared in English and translated to the local language (Afan Oromo) and then retranslated to English to check its consistency. The questionnaire addressed; socio-demographic factors, obstetric related factors, and anemia related factors. The data was collected by 4 diploma nurses and supervised by two MPH supervisors. Data were collected during an exit interview after they got service. The quality of data was assured through training of data collectors and supervisors for two days, pretesting data collection tools on 5% of the sample, and close supervision of the data collection process.

### Data processing and analysis

Data were coded, entered into Epi data version 3.1, and exported to SPSS version 20.0 for analysis. Descriptive statistics were computed to determine the frequency and percentages. Binary logistic regression was conducted and COR with 95% CI was estimated to select the candidate variables for the final model. Then, variables with a *p*-value of < 0.25 at binary logistic regression were taken into a multivariable logistic regression to control confounding. Hosmer-Lemeshow goodness-of-fit with stepwise (backward elimination) logistic regression was used to test for model fitness, and the exact value was 0.078. AOR with 95% CI was estimated to assess the presence of association at multivariable logistic regression. Finally, variables with a p-value of < 0.05 were considered as statistically significant predictors of maternal anemia.

## Results

### 5.1 Socio-demographic characteristics of the study respondents

A total of 286 pregnant women attending antenatal care were included in the study making a response rate of 100%. About 156(54.5%) of the study participants were found in the age group of 20–30. The majority, 264(92.3%) of the respondents were married and most, 114(39.9%) of them were protestant religious followers, followed by orthodox 107(37.4%), and 102(35.7%) of the respondents attended above the secondary school. Oromo ethnicity was the most widely distributed ethnic group in this study area with a frequency of 244 (85.3%) (Table 1).

**Table 1:** Socio- demographic characters of pregnant women attending ANC at WURH, West Ethiopia, 2019

Variable	Category	Frequency	Percent (%)
**Age**	20–30	156	54.5
	30–40	85	29.7
	> 40	45	15.7
**Marital status**	Married	264	92.3
	Single	6	2.1
	Divorced	7	2.4
	Widowed	7	2.4
	Separated	2	0.7
**Religion**	Orthodox	107	37.4
	Protestant	114	39.9
	Muslim	59	20.6
	Catholic	6	2.1
**Occupation**	Housewife	58	20.3
	Employed	76	26.6
	Farmer	75	26.2
	Daily labor	23	8
	Merchant	54	18.9
**Ethnicity**	Oromo	244	85.3
	Amara	36	12.6
	Gurage	6	2.1
**Monthly income (Ethiopian birr)**	< 1000	11	3.8
	1000–2000	129	45.1
	2000–4000	114	39.9
	> 4000	32	11.2
**Educational status**	Uneducated	59	20.6
	Read and write only	29	10.1
	Primary	36	12.6
	Secondary	60	21
	Above secondary	102	35.7

### Reproductive characteristics of respondents

All (100%) of respondents had latrine and more than two-third, 202(71%) of respondents were multigravida. The majority, 210 (73.4%) of women with multigravida gave birth within an interval of < 2 years. Around half of the respondents, 120 (42%) were in the second trimester, while 86 (30.1%) of them were in the third trimester. About 177 (61.9%) of them used contraceptive methods previously. Currently, more than-two third, 221 (77.3%), of pregnant women had taken iron supplementation and 63(22%) had a history of previous abortion. Around 32 (11.2%) of the respondents had a parasitic infection and all of them took medication for it. About 3(1%) of the respondents had a history of chronic diseases (Table 2).

**Table 2:** Clinical and reproductive history of pregnant women attending ANC at WURH, western Ethiopia, 2019

Variable	Category	Frequency	Percent (%)
**Gravidity**	Primigravida	83	29
	Multigravida	203	71
**Birth interval**	< 2 years	210	73.4
	> 2 years	76	26.6
**Gestational age**	First trimester	80	28
	Second trimester	120	42
	Third trimester	86	30.1
**Use of contraceptives**	Yes	177	61.9
	No	109	38.1
**Iron supplementation on current pregnancy**	Yes	221	77.3
	No	65	22.7
**Previous history of abortion**	Yes	62	22
	No	223	78
**Parasitic infection**	Yes	32	11.2
	No	254	88.8
**Presence of chronic diseases**	Yes	3	1
	No	283	99

### Laboratory findings

The hemoglobin level of pregnant who women attended ANC at Wollega University Referral Hospital was checked. Accordingly, the majority 235 (82.2%) of them had hemoglobin levels above 11 g/dl. The result from the stool examination revealed that about 26 (9.1%) of them were found to be positive for different parasites. About 4(1.4%) of the respondents were found to be positive for malarial parasites. Few, 45 (15.7%) of the respondents had a history of malarial attacks in the past 12 months (Table 3).

**Table 3:** Laboratory result of pregnant women attending ANC at WURH, Western Ethiopia, 2019

Variable	Category	Frequency	Percent (%)
**Hemoglobin level**	≤ 7	8	2.8
	8–9.9	24	8.4
	10–11	19	6.6
	> 11	235	82.2
**Stool examination**	Yes	26	9.1
	No	260	90.9
**Blood film**	Malaria parasite present	4	1.4
	Malaria parasite absent	282	98.6
**Malarial attack within 12 months**	Yes	45	15.7
	No	241	84.3

### Prevalence and severity of Anemia among respondents

**Fig. 1:**
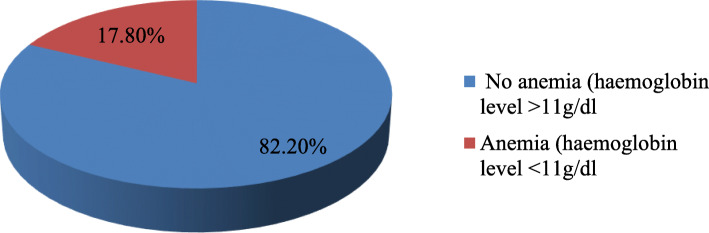
Prevalence of anemia among pregnant women attending ANC at WURH, Western Ethiopia, 2019

**Fig. 2:**
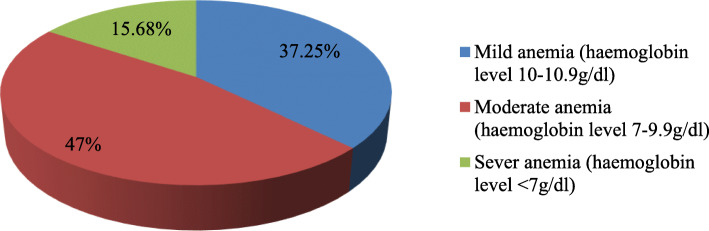
Severity of anemia among anemic pregnant women attending ANC at WURH, Western Ethiopia, 2019

In this study, the overall prevalence of anemia using a cut off level of hemoglobin < 11 g/dl was 51 (17.8%) (Fig. 1). Out of all anemic pregnant women, about 19 (37.25%) were mildly anemic, 24 (47%) were moderately anemic and 8 (15.68%) were severely anemic (Fig. 2).

### Predicting factors of anemia among pregnant women

According to bivariate analysis result; age, occupation, birth interval, parasitic infestation during pregnancy, history of malarial attack in the past 12 months, and iron supplementation during pregnancy were significantly associated with maternal anemia.

After controlling the effect of confounding, multivariable logistic regression revealed that birth interval of less than 2 years (AOR = 2.56 CI [2.84–4.52]), history of malarial attack in the past 12 months (AOR = 2.585[1.181–5.656]) and engaging into daily laborer occupation (AOR = 8.33[2.724–25.497]) showed significant association with maternal anemia.

Pregnant women who had a birth interval of less than 2 years were 2.56 times more likely to be anemic compared to those who had birth intervals of greater than or equal to 2 years (AOR = 2.56 CI [2.84–4.52]). In this study, having a history of malarial attack in the past 12 months shows a significant association with maternal anemia. Pregnant women who had a history of malarial attacks in the past 12 months were 2.6 times more likely to be anemic compared to their counterparts (AOR = 2.585[1.181–5.656]). Similarly, engaging in daily laborer occupations shows a significant association with maternal anemia. Pregnant women who engaged in daily laborer occupations were 8.33 times more likely to be anemic compared to those who engaged in merchant occupation (AOR = 8.33[2.724–25.497]) (Table 4).

**Table 4:** Multivariate logistic regression analysis of factors associated with anemia among pregnant women attending ANC at WURH, Western Ethiopia, 2019

Variable	Category	Anemia		COR (95%)	AOR (95%)
		Yes	No		
**Age**	20–30	26	130	1	1
	31–40	9	76	0.938 [0.106–8.287]	2.145 [0.90–3.150]
	> 40	16	29	0.201 [0.023–1.736]	1.660 [0.32–2.880]
**Birth interval (in year)**	< 2	46	164	3.98 [1.973–8.724]	2.56 [2.84–4.52]*
	> 2	5	71	1	1
**Parasite infection**	Yes	11	21	2.8 [0.16–0.797]	2.211 [0.896–5.461]
	No	40	214	1	1
**Malarial attack in the past 12 months**	Yes	19	26	4.77 [1.113–5.656]	2.585 [1.181–5.656]*
	No	32	209	1	1
**Iron supplementation**	Yes	28	193	1	1
	No	23	42	0.260 [0.17–0.997]	0.542 [0.251–1.40]
**Occupation**	House wife	8	50	0.347 [0.551–7.165]	1.987 [0.551–7.165]
	Employed	4	72	0.50 [0.569–10.923]	2.492 [0.569–10.903]
	Farmer	30	45	4.167 [0.179–3.230]	0.760 [0.179–3.230]
	Daily labor	5	18	1.736 [0.039–0.367]	8.33 [2.724–25.497]*
	Merchant	4	50	1	1

Key: * = significant association at *P*-value < 0.05

## Discussion

This study was conducted with the intention of assessing the prevalence of anemia and identifying its determinants. To describe the prevalence of anemia, WHO criteria to diagnose anemia among pregnant women was used. According to findings from this study the prevalence of anemia among studied pregnant women attending ANC was 51 (17.8%). This finding was found to be comparable with results obtained from studies conducted in Gondar [11], Tikur Ambessa specialized hospital [12], and Benchimaji, Keffa, and Shekka zone public hospitals [13] where the prevalence of anemia was 16.6, 21.3, and 19% respectively. However, this result was lower than the study conducted in Boditti Health center with a prevalence of 60% [14] and Kenya where the prevalence of anemia was 57% [15]. This difference might be due to the study period and the attention given for focused antenatal care and supplementation of iron sulfate throughout the pregnancy. The finding of the current study was higher than the study conducted in Adigrat General Hospital, which was 7.9% [16]. The reason for this discrepancy might be due to differences in methodology including sampling technique among these studies. Also, it may be a result of improvement in health service and the time gap between these studies.

Multivariable logistic regression analysis revealed that Birth interval of less than 2 years, engaging into daily laborer occupations, and having a history of malarial attacks in the past 12 months showed significant association with maternal anemia.

The prevalence of anemia was seen to be increased with close birth interval. Pregnant women who had close birth intervals (less than 2 years) were 2.56 times more likely to be anemic compared to those who had greater than or equal to 2 years of birth intervals. This finding is consistent with a study conducted in Arba Minch [17], a study conducted in Bangladesh [18], a study conducted in Mogadishu [19], and a study conducted in Walayta Soddo, Otona Hospital [20]. This might be related to decreased iron stores of women due to the occurrences of pregnancy in quick succession between subsequent pregnancies. However, the study done at Trinidad and Tobago indicated a birth interval had no association with the prevalence of anemia [21]. The reason for this might be due to limited cases with a birth interval in the above study as well as due to methodological difference.

Having a history of malarial attacks in the past 12 months revealed to have a significant association with maternal anemia. Accordingly, women who had a history of malarial attacks in the past 12 months were 2.6 times more likely to be anemic as compared to their counterparts. This finding is consistent with a study conducted in Mizan Tepi University Teaching Hospital, in which women who had a history of malarial attacks were eight times more likely to be anemic as compared to those having no malarial attack [9], a study conducted in Walayta Soddo, Otona Hospital [20], a study conducted in Azezo health center of Gondar town [22] and study conducted in public Hospitals of Ilu Aba Bora [23]. This might be due to malaria causes anemia by destroying red blood cells at a rate faster than the body can replace them.

In this study, the prevalence of anemia was Eight times higher in those women who led their lives with daily labor as compared to merchants. This finding is consistent with the study conducted in India [24], a study conducted in eastern Nepal [25], and a study conducted in Arba Minch town, Gamo Gofa zone, Ethiopia [17], and a study conducted in Walayta Soddo, Otona Hospital [20]. This might be due to low income that results in a lack of adequate nutrition and poor personal hygiene which exposes them to parasitic infestations.

### Strength and limitation of the study

#### Strength of the study

- The diagnosis of anemia was based on laboratory analysis and did not depend on only clinical assessment.

#### Limitation of the study

- The Cross-sectional nature of the study limits the cause and effect relationship.
- There may be recall bias while subjects were requested to give information about monthly income.

## Conclusion and recommendations

The overall prevalence of anemia in this study using a cut off level of hemoglobin of < 11 g/dl was 17.8% and the majority of them were moderately anemic (Hb level from 8 to 9.9 g/dl). Less than 2 years of birth interval, having a history of malarial attacks in the past 12 months, and engaging in daily laborer occupation were factors which were significantly associated with maternal anemia.

Based on the result of this study the following recommendations were forwarded.

- Information on the importance of birth spacing and contraceptive service information and service should be provided for pregnant women.
- Concerned stakeholders (health professionals working at hospitals and health centers found in the study area) should work on the prevention of malarial attacks, through giving information about prevention methods and provision of an insecticide-treated bed net, particularly for pregnant women and children.
- Concerned bodies (Government and other stakeholders) should work in collaboration to economically empower women to prevent their engagement in risky works including a daily labor.
- Further research on risk factors of anemia which include micronutrient deficiencies, as well as laboratory studies should be conducted to identify the root cause of underlying problems in the pregnant women to guide the health care provider to alleviate the existing problem.

## Data Availability

The data collected for this study can be obtained from the corresponding author based on a reasonable request.

## Abbreviations

ANC: Antenatal care
AOR: Adjusted odd ratio
AIDS: Acquired immune deficiency syndrome
COR: Crude odd ratio
CI: Confidence interval
G/DL: Gram per deciliter
Hb: Hemoglobin
HIV: Human immunodeficiency virus
MCH: Maternal and child health
NRH: Nekemte Referral Hospital
SPSS: Statistical package for social sciences
WHO: World health organization
WURH: Wollega University Referral Hospital

## References

[1] Haidar JA, Pobocik RS. Iron deficiency anemia is not a rare problem among women of reproductive ages in Ethiopia: a community-based cross sectional study. BMC Blood Disord. 2009;8:1–8. 10.1186/1471-2326-9-7 PMC2749016 19735547

[2] Mclean E, Cogswell M, Egli I, Wojdyla D, De BB. Worldwide prevalence of anaemia, WHO Vitamin and Mineral Nutrition Information System, 1993–2005; 2008. 10.1017/S1368980008002401 18498676

[3] Banjari I. Iron deficiency anemia and pregnancy. 2016. 10.5772/intechopen.69114.

[4] Kozuma S. Approaches to Anemia in Pregnancy. J Japan Med Assoc. 2009;137(6):214–218.

[5] Tesfaye DJ, Beshir WG, Dejene T, Tewelde T (2015). Prevalence of intestinal Helminthiases and associated factors among pregnant women attending antenatal Clinic of Nigist Eleni Mohammed Memorial Hospital, Hossana.

[6] Micronutrient deficiencies, iron deficiency anemia. html who 2012. http://www.who.int/nutrition/topics/ida/en/index. Accessed 23 Oct 2019.

[7] Cusick SE, Mei Z, Freedman DS, Looker AC, Ogden CL, Gunter E. Unexplained decline in the prevalence of anemia among US children and women between 1988–1994 and 1999–2002 1–3. 2008;(1);1611–7. 10.3945/ajcn.2008.25926 19064522

[8] Gies S, Yassin MA, Cuevas LE. Comparison of screening methods for anaemia in pregnant women in Awassa , Comparison of screening methods for anaemia in pregnant women in Awassa, Ethiopia. 2003;(January 2019). 10.1046/j.1365-3156.2003.01037.x 12667148

[9] Zekarias B, Meleko A, Hayder A, Nigatu A, Yetagessu T (2017). Prevalence of Anemia and its associated factors among pregnant women attending antenatal care (ANC) in Mizan Tepi University teaching hospital, South West Ethiopia.

[10] Western Oromia, Statistical Agency (2018). Nekemte City office eastern Wollega.

[11] Mulugeta Melku, Zelalem Addis, Meseret Alem,and Bamlaku Enawgaw, Prevalence and predictors of maternal Anemia during pregnancy in Gonder, North west Ethiopia:An institution based cross-sectional study. 2014. 10.1155/2014/108593 PMC3942101 24669317

[12] Jufar AH, Zewde T (2013). Prevalence of Anemia among Pregnant Women Attending Antenatal Care at Tikur Anbessa Specialized Hospital, Addis Abeba,Ethiopia.

[13] Gudeta TA, Regassa TM, Belay AS (2019). Magnitude and factors associated with anemia among pregnant women attending antenatal care in bench Maji, Keffa and Sheka zones of public hospitals, southwest, Ethiopia, 2018: a cross -sectional study. PLoS One.

[14] Lelissa D, Yilma M, Shewalem W, Abraha A, Worku M (2015). Prevalence of anemia among women receiving antenatal care at boditii health center, southern Ethiopia. Clin Med Res.

[15] Okube OT, Mirie W, Odhiambo E, Sabina W, Habtu M (2016). Prevalence and factors associated with anaemia among pregnant women attending antenatal clinic in the second and third trimesters at pumwani maternity hospital Kenya. Open J Obstet Gynecol.

[16] Brhane B, Fitsum M, Haftom L, Aderajew G, Guesh G, Kebede T, Getachew K, Hadush N, Gebre A (2019). Prevalence of anemia and associated factors among pregnant women in Adigrat general hospital, Tigrai, northern Ethiopia, 2018. BMC Res Notes.

[17] Alemayehu Bekele, Marelign Tilahun, and Aleme Mekuria: Prevalence of Anemia and its associated factors among pregnant women attending antenatal Care in Health Institutions of Arba Minch town, Gamo Gofa zone, Ethiopia: A Cross-Sectional Study. 2016. 10.1155/2016/1073192 PMC4779815 27022481

[18] Ahmed S, Mamun M, Mahmud N, Farzana N, Sathi M, Biswas B, Datta A, Ahmad T (2019). Prevalence and associated factors of Anemia among pregnant women receiving antenatal care (ANC) at Fatima Hospital in Jashore, Bangladesh: a cross-sectional study. Food Nutr Sci.

[19] Hassan D, Abdullahi H (2017). Prevalence of Anemia and Its Associated Factors among Pregnant Women Attending Antenatal Clinic at SOS Hospital in Heliwa District, Mogadishu. Adv Soc Sci Res J.

[20] Lealem G., Asrat A., Yaregal A., Andualem M. Anemia and associated factors among pregnant women attending Antenatal Care Clinic in Wolayita Sodo town, Southern Ethiopia, 2015. Ethiop J Health Sci. Vol. 25, No. 2. 10.4314/ejhs.v25i2.8 PMC4478267 26124623

[21] Uche-Nwachi EO, Odekunle A, Jacinto S, Burnett M, Clapperton M, David Y, Durga S, Greene K, Jarvis J, Nixon C, Seereeram R, Poon-King C, Singh R. Anaemia in pregnancy: associations with parity, abortions and child spacing in primary healthcare clinic attendees in Trinidad and Tobago. 2010;10(1):66–70. PMC2895803 20811527

[22] Meseret A, Bamlaku E, Aschalew G, Tigist K, Mohammed S, Yadessa O (2013). Prevalence of anemia and associated risk factors among pregnant women attending antenatal care in Azezo health center Gondar town, Northwest Ethiopia. J Interdiscipl Histopathol.

[23] Adamu K., Efrem N., Lemi B., and Negash W. Magnitude of Anemia and Associated Factors among Pregnant Women Attending Antenatal Care in Public Hospitals of Ilu Abba Bora Zone, South West Ethiopia: A Cross-Sectional Study. Hindawi Anemia Volume 2018, Article ID 9201383, 7 doi: 10.1155/2018/9201383. 10.1155/2018/9201383 PMC6257898 30538862

[24] K Mujibur R., K Mohamed A., S Vijayalakshmi, S Ramkumar, Gulrukh H. Prevalence of Iron Deficiency Anaemia and its Associated Factors among Reproductive Age Women in a Rural Area of Karaikal, Puducherry, India. Journal of Clinical and Diagnostic Research. 2019 Mar, Vol-13(3): LC06-LC10.

[25] Maskey M, Jha N, Poudel S, Yadav D (2014). Anemia in pregnancy and its associated factors: a study from eastern Nepal. Nepal J Epidemiol.

